# Low-Altitude Windshear Estimation Method Based on Four-Dimensional Frequency Domain Compensation for Fuselage Frustum Conformal Array

**DOI:** 10.3390/s23010371

**Published:** 2022-12-29

**Authors:** Hai Li, Lei Zheng, Fanwang Meng

**Affiliations:** 1 Tianjin Key Lab for Advanced Signal Processing, Civil Aviation University of China, Tianjin 300300, China; 2 AVIC Lei Hua Electronic Technology Research Institute, Wuxi 214063, China

**Keywords:** weather radar, low-altitude windshear estimation, fuselage frustum conformal array, target detection, space–time adaptive processing, clutter suppression

## Abstract

In this paper, a low-altitude wind speed estimation method based on the fuselage frustum conformal array system is proposed. Firstly, based on the signal model of the fuselage conformal array radar, the four-dimensional joint phase compensation of the echo data in the Doppler domain and three-dimensional space-frequency domain is performed by using the four-dimensional frequency domain compensation method. Secondly, the clutter covariance matrix is estimated by the compensated echo data, and a space-time Adaptive Processing (STAP) processor suitable for low-altitude windshear target is constructed to suppress clutter. Finally, the maximum Doppler value of each distance cell is extracted, and the wind velocity is estimated. Simulation results show that the proposed method can effectively suppress clutter and accurately estimate wind speed.

## 1. Introduction

Low-altitude windshear is an atmospheric phenomenon in which the airflow below 600 m in height hits the ground and spreads in all directions [1]. Sudden generation, short duration and high hazard are the main characteristics of low-altitude windshear [2]. When an aircraft encounters low-altitude windshear during takeoff and landing phases, the pilot lacks sufficient time and space to control the aircraft’s attitude, which often results in tragic air accidents [3]. Therefore, the study of low-altitude windshear detection and early warning technology is an important research topic in the field of civil aviation. Wind speed estimation is the basis of the entire process of low-altitude windshear detection, which directly affects the accuracy of windshear detection, so it is crucial to estimate wind speed accurately [4,5,6].

Airborne weather radar is an important tool for pilots to detect weather phenomena ahead of the aircraft during flight missions, and pilots can avoid severe weather phenomena such as low-altitude windshear on time based on the display and alerts from the airborne weather radar to ensure the safety of the entire mission [7]. With the development of aviation and the improvement of people’s safety awareness, the practical use of aviation safety demands increasingly high requirements for the functionality and performance of airborne weather radar. At present, the widely used planar array antenna has the defects of limited scanning range and low spatial resolution of radar [8], while the conformal array antenna has the advantages of high space utilization, no limitation of scanning range, large antenna aperture and high azimuthal resolution [9]. Therefore, the adoption of airborne conformal array weather radar is of great significance for weather target detection. At the same time, the conformal array antenna element matches the radar carrier shape, which is in line with the “ blended-wing-body fusion” technology as the main design concept of the next-generation civil aircraft [10,11,12].

When an airborne weather radar detects a low-altitude windshear target ahead, the widely distributed and strong ground clutter tends to drown out the low-altitude windshear signal, making it difficult to achieve wind speed estimation, so clutter suppression before wind speed estimation is especially important. In the forward-looking working state of airborne weather radar, the clutter distribution characteristics of the echo signal of each distance unit vary with the change of distance unit, especially in the near-ground distance unit performance is more dramatic, this phenomenon is called clutter range dependence. The inhomogeneity of clutter caused by range dependence makes the echo signal data samples of each distance unit no longer satisfy the independent identically distributed condition, which causes the deterioration in the performance of traditional clutter suppression methods and the degradation in target detection performance. At present, the compensation methods used for the clutter inhomogeneity problem are mainly Doppler compensation type methods, such as the DW method [13], the HODW method [14,15], the ADC method [16], etc. The DW method and HODW method start from the same point. Both of them are to compensate for the clutter Doppler frequency difference caused by different pitch angles between the reference distance unit and the distance unit under detection so that the clutter in the samples is approximately uniform. The difference between the two is that the DW method only compensates for the Doppler frequency in the main beam direction, while the HODW method compensates for the Doppler frequency corresponding to multiple azimuthal directions. The ADC method compensates the main clutter Doppler difference between the echo data sample of reference distance unit and the distance unit under detection in addition to the main clutter spatial frequency difference. These methods are all proposed based on the conventional uniform line array or planar array airborne radar cases. Conformal array antenna elements are distributed in two-dimensional or three-dimensional surface in space, and different from conventional arrays, its array structure leads to more complex clutter distribution and more serious clutter inhomogeneity [17]. None of the above methods can be applied to the compensation of spatial frequency for a conformal array distributed on a two-dimensional or three-dimensional surface in space. At present, there are literature studies [18,19,20] on low-altitude windshear detection technology, but all of them are for line or planar arrays. There is no research analysis on low-altitude windshear detection technology for fuselage frustum conformal array weather radar in the literature.

A method is proposed to estimate the wind speed of low-altitude windshear in the fuselage frustum conformal array system for the problem that the useful signal will be affected by strong ground clutter when the low-altitude windshear is detected by fuselage frustum conformal array weather radar. The method is based first on the essential reason for the difficulty of clutter suppression caused by the special array structure of the fuselage frustum conformal array, and then uses the four-dimensional frequency domain compensation method for joint phase compensation of the echo data of each distance unit in the Doppler domain and the three-dimensional space-frequency domain. Secondly, the compensated echo data samples are used to estimate the clutter covariance matrix and construct a Space-Time Adaptive Processing (STAP) processor for low-altitude windshear target to accumulate windshear signals while adaptively suppressing ground clutter. Finally, the maximum Doppler value of each distance unit is extracted to achieve the accurate estimation of wind field velocity. The simulation results show that the method can effectively achieve ground clutter suppression and accurate wind speed estimation.

## 2. Fuselage Frustum Conformal Array Signal Model

### 2.1. The Echo Signal Model

**Figure 1:**
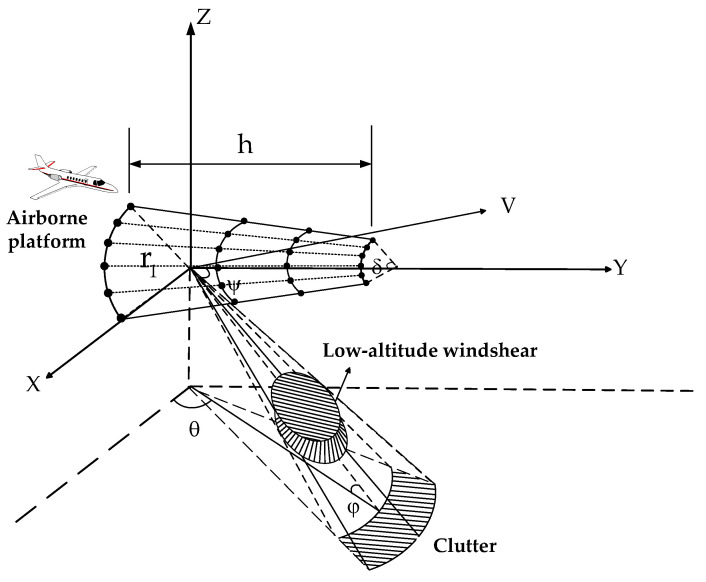
Fuselage frustum conformal array radar model.

Figure 1 shows the model diagram of detecting low-altitude windshear in the forward-looking working state of the fuselage frustum conformal array radar. The angle between the antenna beam and the positive direction of the X axis is defined as the azimuth angle, which is denoted as θ, the pitch angle is denoted as φ, the space cone angle is denoted as ψ, and the aircraft speed is V. The frustum array antenna is distributed on the front fuselage side of the circular profile carrier, with a total of M rows and N columns of array elements.

When the fuselage frustum conformal array detects the low-altitude windshear target, the echo mainly consists of three parts: ground clutter signal $C_{l}$, low-altitude windshear signal $S_{l}$, and noise $\sigma_{l}$. Let $X_{l}$ denote the radar echo of the lth(l=1,2,…,L) distance unit of the fuselage circular platform conformal array, then we have:

(1) $$X_{l}=C_{l}+S_{l}+\sigma_{l},$$

Assuming that the number of transmitted pulses in one coherent processing time of the radar is K, the space-time two-dimensional data received by the lth(l=1,2,…,L) distance cell can be expressed as:

(2) $$X_{l}=\left[\begin{array}{cccccc} x_{l}(1,1) & x_{l}(1,2) & \cdots & x_{l}(1,k) & \cdots & x_{l}(1,K)\\ x_{l}(2,1) & x_{l}(2,2) & \cdots & x_{l}(2,k) & \cdots & x_{l}(2,K)\\ \vdots & \vdots & & \vdots & & \vdots \\ x_{l}(i,1) & x_{l}(i,2) & \cdots & x_{l}(i,k) & \cdots & x_{l}(i,K)\\ \vdots & \vdots & & \vdots & & \vdots \\ x_{l}(MN,1) & x_{l}(MN,2) & \cdots & x_{l}(MN,k) & \cdots & x_{l}(MN,K) \end{array}\right],$$

where $x_{l}(i,k)$ denotes the received data of the lth(l=1,2,…,L) distance unit to the ith array element at the kth pulse. L is the total number of distance units.

### 2.2. Ground Clutter Signal

**Figure 2:**
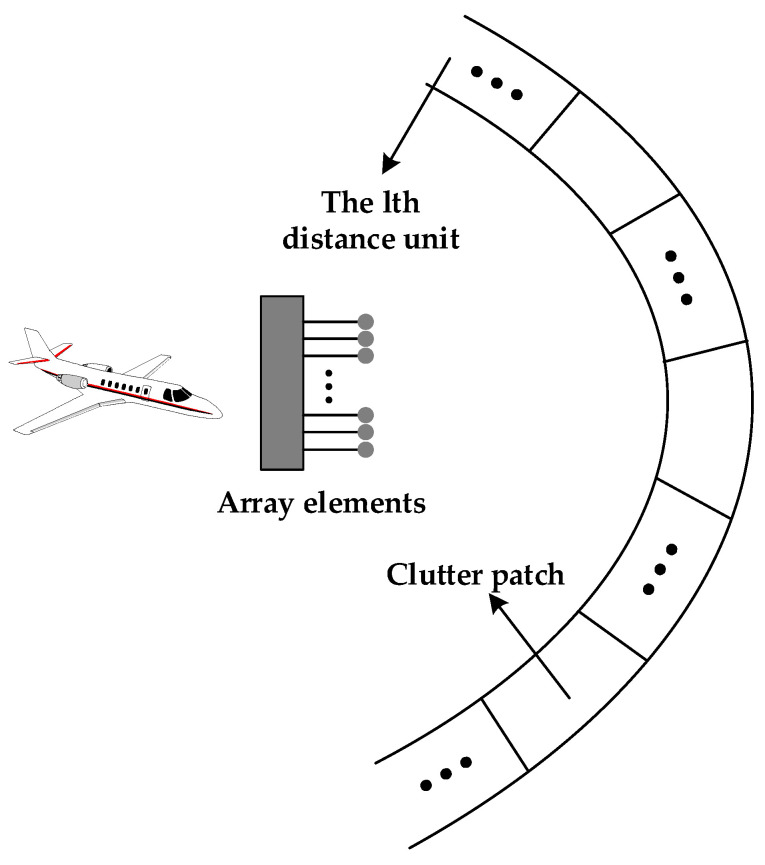
Diagram of Ward model.

The ground clutter signal $C_{l}$ is modeled based on the Ward model [21] developed by the Lincoln Laboratory at MIT. Figure 2 shows the schematic diagram of the Ward model.

Firstly, the width of a single distance unit is determined based on the radar-related parameters of the airborne platform. Then, the horizontal azimuth is divided within the single distance unit, the ground clutter echo signal within the single distance unit is divided into multiple clutter patches, and the echo signal of each clutter patch is calculated. Finally, the echoes of all clutter patches on the single distance unit are summed to obtain the ground clutter echo signal $C_{l}$ of the lth distance unit, and the ground clutter signal is composed of the ground clutter echo signals of all distance units.

Assuming that the ground clutter signal is undulation-free and unambiguous, the ground clutter signal $C_{l}$ is simulated as follows.

Supposing that ϕ(θ,φ) is the beam vector, the geometric relationship from Figure 1 shows that:

(3) $$\phi (\theta ,\varphi )=[\begin{array}{ccc} \cos\theta \cos\varphi & \sin\theta \cos\varphi & \sin\varphi \end{array}],$$

Suppose the radius of the bottom surface of the frustum is $r_{1}$, the height of the frustum is h, and the inclination angle of the frustum is α, the radius of the mth row arc is $r_{m}=r_{1}-\frac{(m-1)h}{M-1}\cot(\alpha )$. The circular angle of the frustum array is δ, and the angle between the angle bisector of the circular angle and the positive direction of the X axis is 45°, which lies in the X−O−Z plane, the position coordinates p(m,n) of the mth row nth column array element can be derived as [22]:

(4) $$p(m,n)=\left\{\begin{array}{l} r_{m}*\cos(\frac{\pi }{2}-\frac{\delta }{2}+(n-1)*\frac{\delta }{N-1})\\ (m-1)*\frac{h}{M-1}\\ r_{m}*\sin(\frac{\pi }{2}-\frac{\delta }{2}+(n-1)*\frac{\delta }{N-1}) \end{array}\right.,$$

The matrix of array element coordinates P is expressed as:

(5) $$P={\left[\begin{array}{ccccccc} p(1,1) & p(1,2) & \cdots & p(1,N) & p(2,1) & \cdots & p(M,N) \end{array}\right]}^{T},$$

From this, the spatial angular frequency $w_{sc}$ of the frustum array clutter can be found and its relation to the cosine of the spatial cone angle cosψ can be deduced as follows:

(6) $$\begin{array}{l} w_{sc}(m,n,\theta ,\varphi_{l})=\frac{2\pi }{\lambda }(\phi (\theta ,\varphi_{l})*p(m,n))\\ \;\;\;=\frac{2\pi }{\lambda }[r_{m}*\sin\left(\delta \left(\frac{1}{2}-\frac{\left(n-1\right)}{N-1}\right)\right)*\sin\varphi_{l}\cos\theta \\ \;\;\;+(m-1)\frac{h}{M-1}*\sin\varphi_{l}\sin\theta +r_{m}*\cos\left(\delta \left(\frac{1}{2}-\frac{\left(n-1\right)}{N-1}\right)\right)*\cos\varphi_{l}]\\ \;\;\;=\frac{2\pi }{\lambda }[r_{m}*\sin\left(\delta \left(\frac{1}{2}-\frac{\left(n-1\right)}{N-1}\right)\right)*\cos\psi_{l}\\ \;\;\;+(m-1)\frac{h}{M-1}*\sqrt{\sin^{2}\varphi_{l}-\cos^{2}\psi_{l}}+r_{m}*\cos\left(\delta \left(\frac{1}{2}-\frac{\left(n-1\right)}{N-1}\right)\right)*\cos\varphi_{l}] \end{array}$$

The time angular frequency $w_{tc}$ is expressed as:

(7) $$w_{tc}(\theta ,\varphi_{l})=\frac{4\pi V}{\lambda f_{r}}\sin\theta \cos\varphi_{l},$$

In which $\varphi_{l}$ is the pitch angle of the lth distance unit, $\psi_{l}$ is the spatial cone angle of the lth distance unit, λ is the wavelength, and $f_{r}$ is the pulse repetition frequency of the radar.

Assuming that the ground clutter reception data of the mth row nth column array element for the lth distance cell at the kth pulse is $C_{l}(m,n,k)$, and the range of azimuth θ is $\left[0,\pi \right]$, then we have:

(8) $$C_{l}(m,n,k)=\frac{1}{R_{l}{}^{2}}\int_{0}^{\pi }F(\theta ,\varphi_{l})e^{jw_{sc}(m,n,\theta ,\varphi_{l})+j(k-1)w_{tc}(\theta ,\varphi_{l})}d\theta ,$$

In which $F(\theta ,\varphi_{l})$ is the antenna orientation map of the frustum array and $R_{l}$ is the radar slope distance corresponding to the lth distance unit.

### 2.3. Low-Altitude Windshear Signal

Low-altitude windshear is a distributed target, and the windshear wind field consists of multiple meteorological scattering particles, so the low-altitude windshear signal echo is a superposition of all scattering point echoes within the radar beam range.

The windshear wind field is modeled by hydrodynamic simulation software [23] to obtain information on the field density and velocity values of the windshear wind field. The scattering point method [24] is adopted to superimpose the low-altitude windshear signal echo $S_{l}$ at each scattering point within each distance unit to simulate the low-altitude windshear wind field. In the case of wet low-altitude windshear, there are a large number of rainfall particles, small droplets, etc. within the wind field. Therefore, for the qth scattering point in the wind field within a single distance unit, the echo signal amplitude can be deduced by the meteorological radar equation according to [23,24] as:

(9) $$A_{q}=\sqrt{\frac{0.93P_{t}G^{2}\pi^{2}Z}{64\lambda^{2}R_{q}{}^{4}}},$$

In which $P_{t}$ is the radar transmitter power, G is the antenna gain, $R_{q}$ is the slope distance between the qth scattering point and the carrier, λ is the radar wavelength, 0.93 is the ion product constant, Z is the wind field reflectivity factor. 

In radar meteorological theory, the equivalent reflectivity factor is often used to measure the echo intensity of a meteorological target. The equivalent reflectivity factor is related to the size, morphology, spatial distribution, phase and density of precipitation particles per unit volume in a meteorological target. Therefore, the characteristics of the equivalent reflectivity factor are used to distinguish the type of meteorological target when detecting meteorological targets. According to [25], the wind field reflectivity factor is expressed as:

(10) $$Z=\frac{10^{18}\times 720{\left(\rho q_{r}\right)}^{1.75}}{\pi^{1.75}N_{r}{}^{0.75}\rho_{r}{}^{1.75}},$$

where ρ is the atmospheric density, $q_{r}$ is the mass ratio of water vapor to air in the atmosphere; $N_{r}$ is a constant, $N_{r}=8\times 10^{6}m^{-4}$,$\rho_{r}$ is the density of water.

Let $S_{l}(m,n,k)$ be the received data of the mth row nth column array element of the frustum array for the lth distance cell windshear wind field at the kth pulse, denoted as:

(11) $$S_{l}(m,n,k)=\sum_{q=1}^{Q}\frac{A_{q}}{R_{q}{}^{2}}e^{jw_{ss}(m,n,\theta_{q},\varphi_{q})+j(k-1)w_{ts}(\theta_{q},\varphi_{q})},$$

where Q denotes the number of scattering points of the wind field within the beam range at a single distance cell, $v_{q}$ is the relative velocity of the qth scattering point to the carrier, $w_{ss}(m,n,\theta_{q},\varphi_{q})$ and $w_{ts}(\theta_{q},\varphi_{q})$ denote the spatial and temporal angular frequency of the fuselage frustum conformal array at the qth scattering point, and we have:

(12) $$\begin{array}{l} w_{ss}(m,n,\theta_{q},\varphi_{q})=\frac{2\pi }{\lambda }*\phi (\theta_{q},\varphi_{q})*p(m,n)\\ w_{ts}(\theta_{q},\varphi_{q})=\frac{4\pi v_{q}}{\lambda f_{r}} \end{array}$$

## 3. Low-Altitude Windshear Speed Estimation Method Based on Four-Dimensional Frequency Domain Compensation Method

Compared with the conventional array type, fuselage frustum conformal array is distributed in space as a three-dimensional surface, and the array structure leads to stronger clutter inhomogeneity, so the conventional Doppler compensation method is no longer applicable, and it is more difficult to deal with clutter suppression for the fuselage frustum conformal array. For the array structure of the fuselage frustum conformal array, this paper first adopts the four-dimensional frequency domain compensation method to compensate for the echo data in the Doppler domain and three-dimensional space-frequency domain. Then, the method uses the compensated echo data samples to estimate the clutter covariance matrix and constructs the optimal space-time processor for clutter suppression. Finally, it extracts the maximum Doppler frequency of each distance unit echo to estimate the wind field velocity. In this method, the covariance matrix estimation based on the four-dimensional frequency domain compensation method and the STAP-based wind speed estimation method are the key steps, which are described separately below.

### 3.1. Covariance Matrix Estimation Based on Four-Dimensional Frequency Domain Compensation Method

In order to address the problem of inhomogeneity in the fuselage frustum conformal array radar, a four-dimensional frequency domain compensation method is proposed. The conventional Doppler compensation method is focused only on the conventional array type for echo compensation, which is unable to take into account the different characteristics of the clutter distribution due to the complex array structure of the fuselage frustum conformal array. The four-dimensional frequency domain compensation method deals with the clutter inhomogeneity problem from the clutter distribution characteristics of the fuselage frustum conformal array. In the next section, we first analyze the clutter characteristics of the fuselage frustum conformal array, then compensate the echo data of the fuselage frustum conformal array by the four-dimensional frequency domain compensation method, and finally use the compensated echo data for covariance matrix estimation.

#### 3.1.1. Analysis of the Clutter Characteristics of the Fuselage Frustum Conformal Array

Assuming that the angle between the direction of flight of the aircraft and the positive direction of the X axis is $\theta_{p}$, and the angle between the positive direction of the Y axis is $\varphi_{p}$, $\dot{V}(\theta_{p},\varphi_{p})$ is the velocity vector in the direction of flight of the aircraft, $\dot{V}(\theta_{p},\varphi_{p})={\left[\begin{array}{ccc} V\cos\theta_{p} & V\sin\theta_{p} & 0 \end{array}\right]}^{T}$. Suppose that the aircraft flies parallel to the ground, which means $\varphi_{p}=0$, and then the normalized Doppler frequency of the ground clutter signal $F_{d}$ can be expressed as:

(13) $$\begin{array}{l} F_{d}=4\phi (\theta ,\varphi )\cdot \dot{V}(\theta_{p},\varphi_{p})/(\lambda f_{r})\\ \;\;\;\;\;=4V(\cos\varphi \cos\theta \cos\theta_{p}+\cos\varphi \sin\theta \sin\theta_{p})/(\lambda f_{r}) \end{array}$$

The normalized space domain frequency $F_{s}$ of the ground clutter signal can be expressed as:

(14) $$F_{s}=\phi (\theta ,\varphi )=[\begin{array}{ccc} \cos\theta \cos\varphi & \sin\theta \cos\varphi & \sin\varphi \end{array}],$$

From Equation (14), it can be seen that the normalized space-frequency $F_{s}$ is distributed in three dimensions. For ease of presentation, define $F_{sx}=\cos\varphi \cos\theta$, $F_{sy}=\cos\varphi \sin\theta$, and $F_{sz}=\sin\varphi$.The $F_{sx}$ domain represents the projection of the space-frequency $F_{s}$ under the X axis, the $F_{sy}$ domain represents the projection of the space-frequency $F_{s}$ under the Y axis, and the $F_{sz}$ domain represents the projection of the space-frequency $F_{s}$ under the Z axis, which means $F_{s}=[\begin{array}{ccc} F_{sx} & F_{sy} & F_{sz} \end{array}]$. As seen in Equations (13) and (14), the clutter spectra of the airborne radar are interacting in the Doppler and space domain.

For the line array antenna, the geometric distribution of the array elements in space is one-dimensional. Assuming that the line array axis is in line with the positive direction of X axis. Then, the direction of variation of the array elements in space is only in the direction of X axis. Therefore, the clutter space-time spectrum of the airborne line array radar can be described in two-dimensional space $F_{d}-F_{sx}$, and its two-dimensional distribution characteristic is obtained from Equation (13) [26]:

(15) $$F_{d-linear}=4V(F_{sx}\cos\theta_{p}+\sin\theta_{p}\sqrt{\cos^{2}\varphi -F_{sx}{}^{2}})/(\lambda f_{r}),$$

For the planar array antenna, the geometric distribution of its array elements in space is two-dimensional. Assuming that the length of the planar array is in line with the positive direction of the X axis and the width is in line with the positive direction of Y axis. Then the variation of the array elements in space is in the X−O−Y plane, so the clutter of the airborne planar array radar is described in three-dimensional space $F_{d}-F_{sx}-F_{sy}$, and its distribution characteristics are obtained from Equation (13) [26]:

(16) $$F_{d-planar}=4V(F_{sx}\cos\theta_{p}+F_{sy}\sin\theta_{p})/(\lambda f_{r}),$$

As for the fuselage frustum conformal array, the antenna array elements are geometrically distributed in space as three-dimensional space, so its clutter distribution characteristic needs to be described in four-dimensional space $F_{d}-F_{sx}-F_{sy}-F_{sz}$, which can be obtained from Equation (13) [26]:

(17) $$F_{d-frustum}=4V\left[\sqrt{1-F_{sz}{}^{2}}(\frac{F_{sx}}{\cos\varphi }\cos\theta_{p}+\frac{F_{sy}}{\cos\varphi }\sin\theta_{p})\right]/(\lambda f_{r}),$$

From Equation (17), it can be seen that the range of values of $F_{sx}$, $F_{sy}$ and $F_{sz}$ varies with different pitch angles $\varphi_{l}$ corresponding to different distance units in the clutter space-frequency of the fuselage frustum conformal array. The Doppler frequency $F_{d-frustum}$ is a function of $F_{sx}$, $F_{sy}$ and $F_{sz}$, and the joint distribution curve of Doppler frequency and space domain of the clutter in different distance units is varied within the four-dimensional space $F_{d}-F_{sx}-F_{sy}-F_{sz}$. It is also known from Equation (15) and Equation (16) that the clutter distribution of the line and planar array airborne radar is only distributed in two and three dimensions, and the clutter distribution of fuselage frustum conformal array is more complex in comparison with it. Therefore, the clutter of fuselage frustum conformal array radar has more serious inhomogeneity in comparison with the line or planar array.

**Figure 3:**
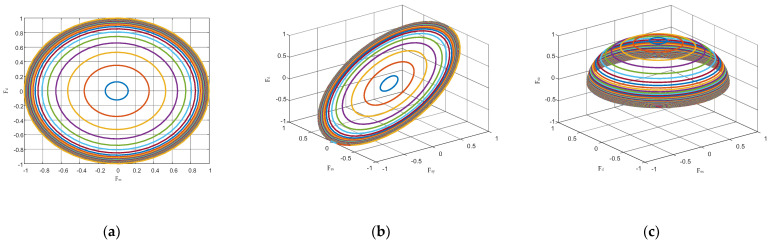
Clutter spectrum distribution for each distance unit. (**a**) F_d_-F_sx_ (**b**) F_d_-F_sx_-F_sy_ (**c**) F_d_-F_sx_-F_sz._

Figure 3 shows the distribution curves of multiple distance unit clutter echo signals of the fuselage frustum conformal array when $\theta_{p}=90{}^{\circ}$. In which, Figure 3a shows the spatial one-dimensional distributed clutter characteristics; Figure 3b,c show the spatial three-dimensional distributed clutter characteristics. Since it is impossible to show the clutter characteristics in the four-dimensional space $F_{d}-F_{sx}-F_{sy}-F_{sz}$ by four-dimensional coordinates, two three-dimensional coordinate systems are used to describe them. From Figure 3, it can be seen that the main clutter position of the fuselage frustum conformal array varies along the four frequency directions of $F_{d}$, $F_{sx}$, $F_{sy}$ and $F_{sz}$ for different main beam pointing cases. The conventional Doppler compensation method only compensates for the clutter in two-dimensional space, $F_{d}-F_{sx}$, and is no longer applicable to the fuselage frustum conformal array. Therefore, it is necessary to perform clutter suppression in four-dimensional space $F_{d}-F_{sx}-F_{sy}-F_{sz}$ for the fuselage frustum conformal array.

#### 3.1.2. Covariance Matrix Estimation Based on Four-Dimensional Frequency Domain Compensation Method

The analysis of the clutter characteristics of the fuselage frustum conformal array shows that the clutter characteristics are determined by the four-dimensional frequency $F_{d}-F_{sx}-F_{sy}-F_{sz}$, and the main clutter position of different distance units is varied in four-dimensional space, while the conventional Doppler compensation method compensates for the clutter spectrum in two-dimensional space $F_{d}-F_{sx}$. Based on this, the four-dimensional frequency domain compensation method extends the two-dimensional space $F_{d}-F_{sx}$ to the four-dimensional space $F_{d}-F_{sx}-F_{sy}-F_{sz}$ to compensate for the spectrum of the fuselage frustum conformal array clutter. The basic principle is to take the center of the clutter spectrum of the distance under detected as the base, move the center of the clutter spectrum of the reference distance unit along the Doppler direction and the three-dimensional beam directions, and make the center of the clutter spectrum of the two coincide after the frequency shift so that completing the compensation of the clutter spectrum.

The four-dimensional frequency domain compensation method requires joint compensation in the four spaces of Doppler domain $F_{d}$, $F_{sx}$ domain, $F_{sy}$ domain and $F_{sz}$ domain, which are discussed separately below.

In the Doppler domain $F_{d}$, to make the reference distance unit coincide with the center of the clutter spectrum of the distance unit under detection, the center of the clutter spectrum of the reference distance unit needs to be shifted along the Doppler frequency axis, and the frequency shift is the difference between the two Doppler frequency.

Assuming that the azimuth of the main beam is $\theta_{0}$, the normalized Doppler frequency $f_{dl}$ at the center of the clutter spectrum of the lth distance cell is:

(18) $$f_{dl}=\frac{2V}{\lambda f_{r}}\sin\theta_{0}\cos\varphi_{l},$$

The normalized Doppler frequency $f_{dt}$ at the center of the clutter spectrum of the tth distance cell is:

(19) $$f_{dt}=\frac{2V}{\lambda f_{r}}\sin\theta_{0}\cos\varphi_{t},$$

To make the tth distance unit coincide with the center of the clutter spectrum of the lth distance unit which is under detection, the center of the clutter spectrum of the tth distance unit needs to be frequency shifted along the Doppler frequency axis towards the center of the clutter spectrum of the lth distance unit by the following amount:

(20) $$\Delta f_{dl}=f_{dl}-f_{dt},$$

Then the compensation matrix $\beta_{dl}$ of the Doppler domain $F_{d}$ is expressed as:

(21) $$\beta_{dl}=\left[\begin{array}{cccc} 1 & 0 & \cdots & 0\\ 0 & e^{j2\pi \Delta f_{dl}} & \cdots & 0\\ \vdots & \vdots & \ddots & \vdots \\ 0 & 0 & \cdots & e^{j2\pi (K-1)\Delta f_{dl}} \end{array}\right],$$

In the space domain $F_{sx}$, to coincide the center of the clutter spectrum of the reference distance unit and the distance unit under detection, the clutter spectrum center of the reference distance unit needs to be shifted along the $F_{sx}$ axis of the space domain frequency, and the frequency shift is the difference between the space domain frequency of the two in the $F_{sx}$ domain.

At this point, the normalized space domain frequency $f_{sxl}$ in the $F_{sx}$ domain for the center of the clutter spectrum of the lth distance unit is shown in the following equation:

(22) $$f_{sxl}=\cos\theta_{0}\cos\varphi_{l},$$

The normalized space domain frequency $f_{sxt}$ in the $F_{sx}$ domain for the center of the clutter spectrum of the tth distance unit is given by the following equation:

(23) $$f_{sxt}=\cos\theta_{0}\cos\varphi_{t},$$

To make the center of the clutter spectrum of the tth distance unit coincide with that of the lth distance unit which is under detection, the center of the clutter spectrum of the $t^{th}$ distance unit needs to be frequency shifted along the $F_{sx}$ axis toward the center of the clutter spectrum of the lth distance unit by the following amount:

(24) $$\Delta f_{sxl}=f_{sxl}-f_{sxt},$$

Then the space domain compensation matrix $\beta_{sxl}$ for the $F_{sx}$ domain is expressed as:

(25) $$\beta_{sxl}=\left[\begin{array}{cccc} 1 & 0 & \cdots & 0\\ 0 & e^{j\frac{2\pi \Delta f_{sxl}}{\lambda }\Delta x_{2}} & \cdots & 0\\ \vdots & \vdots & \ddots & \vdots \\ 0 & 0 & \cdots & e^{j\frac{2\pi \Delta f_{sxl}}{\lambda }\Delta x_{M\times N}} \end{array}\right],$$

In which $\Delta x_{M\times N}$ denotes the difference between the coordinates of the (M×N)th array element and the first array element on the X axis.

In the space domain $F_{sy}$, to coincide the clutter spectrum center of the reference distance unit and the distance unit under detection, the clutter spectrum center of the reference distance unit needs to be shifted along the $F_{sy}$ axis of the space domain frequency, and the frequency shift is the difference between the space domain frequency of the two in the $F_{sy}$ domain.

At this point, the normalized space domain frequency $f_{syl}$ in the $F_{sy}$ domain for the center of the clutter spectrum of the lth distance unit is shown in the following equation:

(26) $$f_{syl}=\sin\theta_{0}\cos\varphi_{l},$$

The normalized space domain frequency $f_{syt}$ in the $F_{sy}$ domain for the center of the clutter spectrum of the tth distance unit is given by the following equation:

(27) $$f_{syt}=\sin\theta_{0}\cos\varphi_{t},$$

To make the tth distance unit coincide with the center of the clutter spectrum of the lth distance unit under detection, the center of the clutter spectrum of the tth distance unit needs to be frequency shifted along the $F_{sy}$ axis toward the center of the clutter spectrum of the lth distance unit by the following amount:

(28) $$\Delta f_{syl}=f_{syl}-f_{syt},$$

Then, the space domain compensation matrix $\beta_{syl}$ for the $F_{sy}$ domain is expressed as:

(29) $$\beta_{syl}=\left[\begin{array}{cccc} 1 & 0 & \cdots & 0\\ 0 & e^{j\frac{2\pi \Delta f_{syl}}{\lambda }\Delta y_{2}} & \cdots & 0\\ \vdots & \vdots & \ddots & \vdots \\ 0 & 0 & \cdots & e^{j\frac{2\pi \Delta f_{syl}}{\lambda }\Delta y_{M\times N}} \end{array}\right],$$

In which $\Delta y_{M\times N}$ denotes the difference between the coordinates of the (M×N)th array element and the first array element on the Y axis.

In the space domain $F_{sz}$, to coincide the clutter spectrum centers of the reference distance unit and the distance unit under detection, the clutter spectrum center of the reference distance unit needs to be shifted along the $F_{sz}$ axis of the space domain frequency, and the frequency shift is the difference between the space domain frequency of the two in the $F_{sz}$ domain.

At this point, the normalized space domain frequency $f_{szl}$ in the $F_{sz}$ domain for the center of the clutter spectrum of the lth distance unit is shown in the following equation:

(30) $$f_{szl}=\sin\varphi_{l},$$

The normalized space domain frequency $f_{szt}$ in the $F_{sz}$ domain for the center of the clutter spectrum of the tth distance cell is given by the following equation:

(31) $$f_{szt}=\sin\varphi_{t},$$

To make the tth distance unit coincide with the center of the clutter spectrum of the lth distance unit which is under detection, the center of the clutter spectrum of the tth distance unit needs to be frequency shifted along the $F_{sz}$ axis toward the center of the clutter spectrum of the lth distance unit by the following amount:

(32) $$\Delta f_{szl}=f_{szl}-f_{szt},$$

Then, the space domain compensation matrix $\beta_{szl}$ for the $F_{sz}$ domain is expressed as:

(33) $$\beta_{szl}=\left[\begin{array}{cccc} 1 & 0 & \cdots & 0\\ 0 & e^{j\frac{2\pi \Delta f_{szl}}{\lambda }\Delta z_{2}} & \cdots & 0\\ \vdots & \vdots & \ddots & \vdots \\ 0 & 0 & \cdots & e^{j\frac{2\pi \Delta f_{szl}}{\lambda }\Delta z_{M\times N}} \end{array}\right],$$

In which $\Delta z_{M\times N}$ denotes the difference between the coordinates of the (M×N)th array element and the first array element on the Z axis.

From Equations (21), (25), (29), and (33), the compensation matrix $\beta_{l}$ for the joint four-dimensional frequency domain $F_{d}-F_{sx}-F_{sy}-F_{sz}$ is expressed as [26,27]:

(34) $$\beta_{l}=\beta_{dl}⊗(\beta_{sxl}⊙\beta_{syl}⊙\beta_{szl}),$$

In which ⊗ is the Kronecker product and ⊙ is the Hadamard product.

Suppose that the echo data of the lth distance unit is $X_{l}$. Then the sample $X_{l}{}^{\prime }$ of the echo data of the lth distance unit after the compensation by the four-dimensional frequency domain compensation method is expressed as:

(35) $$X_{l}^{\prime }=\beta_{l}\cdot X_{l},$$

After compensation, the inhomogeneity of the clutter in the echo data samples caused by the fuselage frustum conformal array structure is improved. 

The data of the lth distance unit echo is selected as the distance unit sample under detection, and the clutter covariance matrix of the distance unit sample under detection is estimated by the reference distance unit echo data samples as:

(36) $${\hat{R}}_{l}=\frac{1}{L}\left(\sum_{j=1;j\neq l}^{L}X_{j}^{\prime }X_{j}^{\prime }{}^{H}+X_{l}X_{l}{}^{H}\right),$$

### 3.2. STAP-Based Wind Speed Estimation Method

The space-time adaptive processor technique is based on space-time coupling characteristics of the echo to perform clutter suppression and signal matching [28], and the optimal processor can be described as a mathematical optimization problem as follows.

(37) $$\left\{\begin{array}{l} \begin{array}{cc} \min & \omega_{l}{}^{H}{\hat{R}}_{l}\omega_{l} \end{array}\\ \begin{array}{cc} st. & \omega_{l}{}^{H}S=1 \end{array} \end{array}\right.,$$

where $\omega_{l}$ is the optimal processor weight vector, ${\hat{R}}_{l}$ is the covariance matrix of the lth distance unit echo data under the fuselage frustum conformal array, $S_{s}$ and $S_{t}$ are the spatial and temporal steering vectors, and S is the space-time two-dimensional steering vector of the fuselage frustum conformal array:

(38) $$S=S_{s}⊗S_{t},$$

(39) $$S_{s}={\left[e^{jw_{s1}},e^{jw_{s2}},\ldots e^{jw_{sMN}}\right]}^{T},$$

(40) $$S_{t}={\left[1,e^{jw_{t}},\ldots e^{j(K-1)w_{t}}\right]}^{T},$$

In which $w_{s}$ is the normalized spatial angular frequency and $w_{t}$ is the normalized temporal angular frequency. At this point, the optimal weight vector of the fuselage frustum conformal array space-time adaptive filter can be derived as follows:

(41) $$\omega_{l}^{}=\mu {\hat{R}}_{l}^{-1}S,$$

Taking the estimated ${\hat{R}}_{l}$ into the above equation, the result of matching the fuselage frustum conformal array can thus be obtained as $y_{l}$:

(42) $$y_{l}=\omega_{l}{}^{H}X_{l},$$

By matching the filtering of target signals in different Doppler bands and solving the output signal power value of the optimal processor, the processor can effectively suppress ground clutter and accumulate windshear signals when the output power is maximum. Then, the Doppler frequency of the distance unit under detection is searched in one dimension, and the estimated Doppler frequency $\overset{\wedge }{f_{l}}$ of the distance unit under detection can be obtained, from which the estimated wind speed $\overset{\wedge }{v_{l}}$ of the distance unit can be calculated as:

(43) $$\overset{\wedge }{v_{l}}=\frac{\lambda \overset{\wedge }{f_{l}}f_{r}}{4},$$

Finally, the echo data of all the distance units of the fuselage frustum conformal array are subjected to optimal space-time adaptive processing, and the wind field velocity is estimated, so that the variation of the low-altitude windshear wind speed can be derived for different distance units.

### 3.3. Method Flow

**Figure 4:**
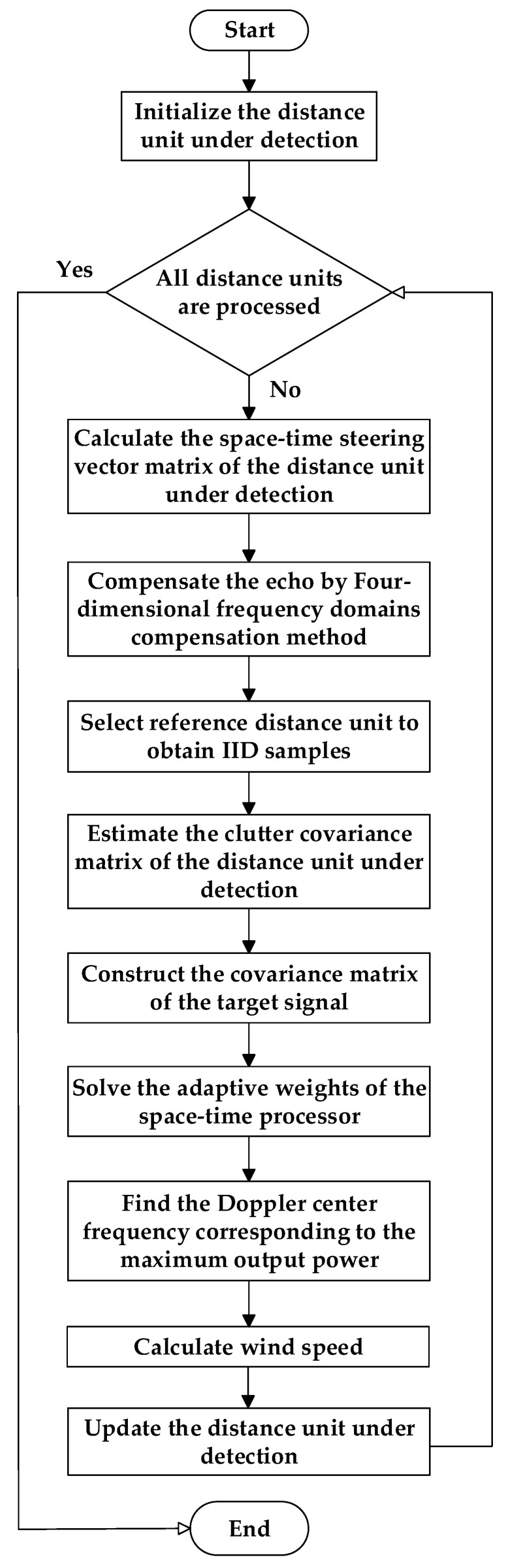
The basic flowchart of the low-altitude windshear estimation method under the fuselage frustum conformal array structure.

Figure 4 shows the basic flowchart of the low-altitude windshear wind speed estimation method under the fuselage frustum conformal array structure. The method uses a four-dimensional frequency domain compensation method to compensate for the echo data samples to remove the clutter inhomogeneity caused by the structure of the frustum array. Then it uses the compensated echo data to estimate the clutter covariance matrix and constructs the optimal space-time processor for clutter suppression. Finally, it extracts the maximum Doppler frequency of each distance unit and estimates the low-altitude windshear wind field wind speed. The specific steps of processing the echo data are as follows.

Step 1: Compensate the echo data by four-dimensional frequency domain compensation method to remove the inhomogeneity of ground clutter caused by the structure of the fuselage frustum conformal array;

Step 2: Estimate the clutter covariance matrix of the distance unit under detection from the echo data samples of the reference distance unit;

Step 3: Construct the space-time adaptive processor, solve the optimal weight vector of the processor, complete clutter suppression and signal matching, estimate the center frequency of the low-altitude windshear signal within the present distance unit, then get the wind speed estimation result;

Step 4: Update the distance unit under detection and deal with the echo data samples of all distance units within the radar operating range to get the final wind speed estimation result.

## 4. Simulation Results and Analysis

### 4.1. Simulation Condition Settings

The values of the main simulation parameters of the radar system are shown in Table 1.

### 4.2. Analysis of Simulation Results

**Figure 5:**
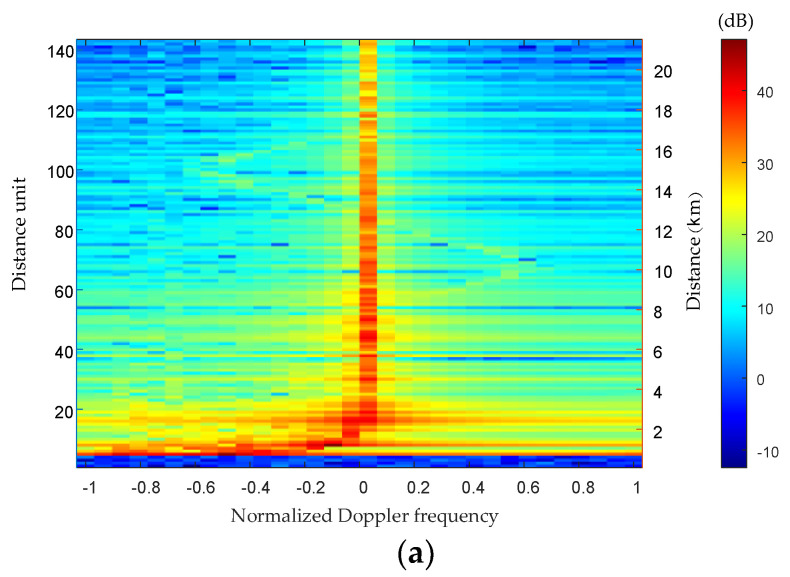

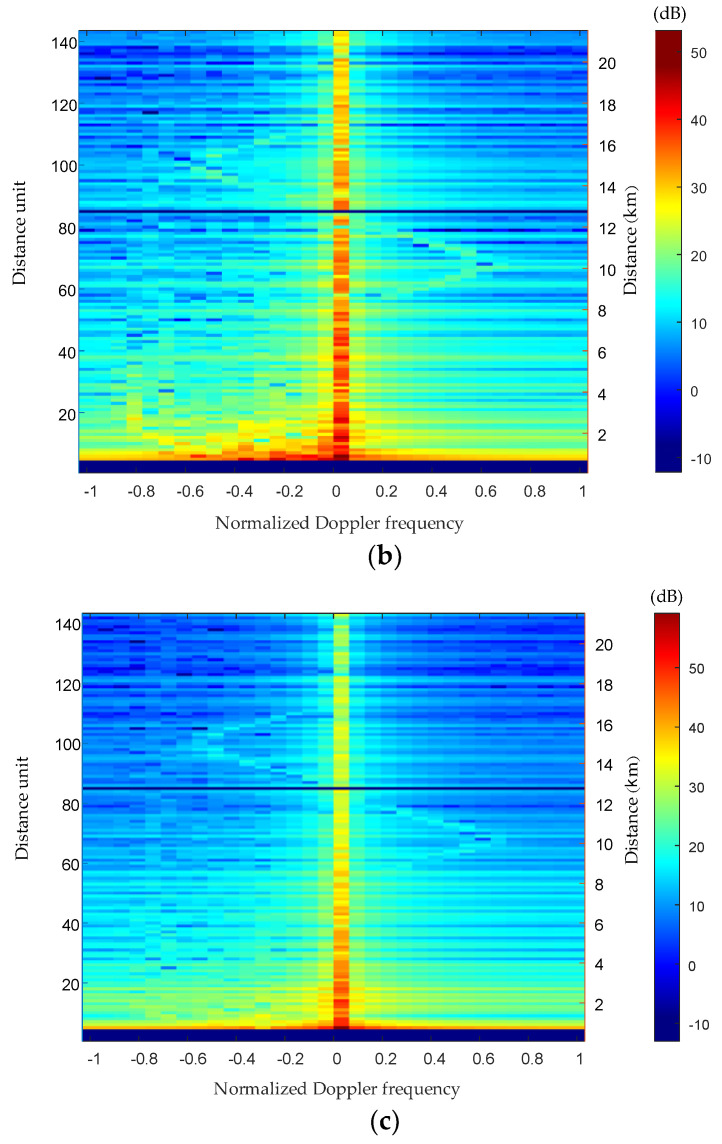
Comparison of Distance-Doppler spectra before and after compensation of fuselage frustum conformal array. (**a**) before compensation (**b**) after DW compensation (**c**) this paper.

Figure 5 shows the Distance-Doppler spectra of the echo signal before and after the compensation of the fuselage frustum conformal array. Among them, Figure 5a shows the Distance-Doppler spectrum of the echo signal in the uncompensated case of the fuselage frustum conformal array. Figure 5b shows the Distance-Doppler spectrum of the echo signal after the DW compensation method. Figure 5c shows the Distance-Doppler spectrum of the echo signal after the compensation by the four-dimensional frequency domain compensation method proposed in this paper.

As can be seen from Figure 5a, the ground clutter signal is mainly concentrated on the zero frequency, and its amplitude is significantly higher than that of the windshear signal, even drowning the windshear signal. The Distance-Doppler spectrum of the ground clutter signal in the near-ground distance unit shows a serious range dependence, which increases the difficulty of the subsequent clutter suppression processing. Compared with Figure 5a, the range dependence of the near-ground distance unit has been suppressed to some extent in Figure 5b after compensated by the DW compensation method. However, the clutter amplitude is still much higher than that of the windshear signal, which is submerged in the ground clutter signal, making it difficult to estimate the wind speed.

After compensating the echo signal by the four-dimensional frequency domain compensation method proposed in this paper, the clutter range dependence of the near-ground distance unit has been suppressed in Figure 5c. The method reduces the variability of the clutter space-time distribution between the echo data samples due to the structure of the fuselage frustum conformal array. The windshear signal can be clearly distinguished from the ground clutter, which is easier for the subsequent wind speed estimation.

**Figure 6:**
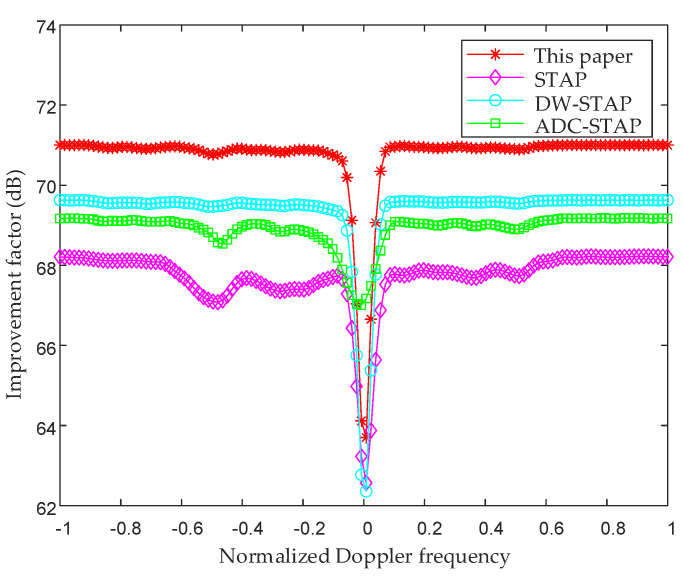
Comparison chart of improvement factors for the 80th distance unit.

Figure 6 shows the comparison of the improvement factors of the method in this paper, the STAP method, the DW-STAP method, and the ADC-STAP method (80th distance unit). From the figure, it can be seen that all of the above methods have performance notches in the Doppler main clutter region since the main clutter is located at zero frequency and the performance is lost. Compared with other methods, the performance notch formed by the method in this paper is narrower and shallower in the region of the main clutter. This shows that this method can be more effective for ground clutter suppression in the fuselage frustum conformal array system, which is the basis for the subsequent low-altitude windshear wind speed estimation.

**Figure 7:**
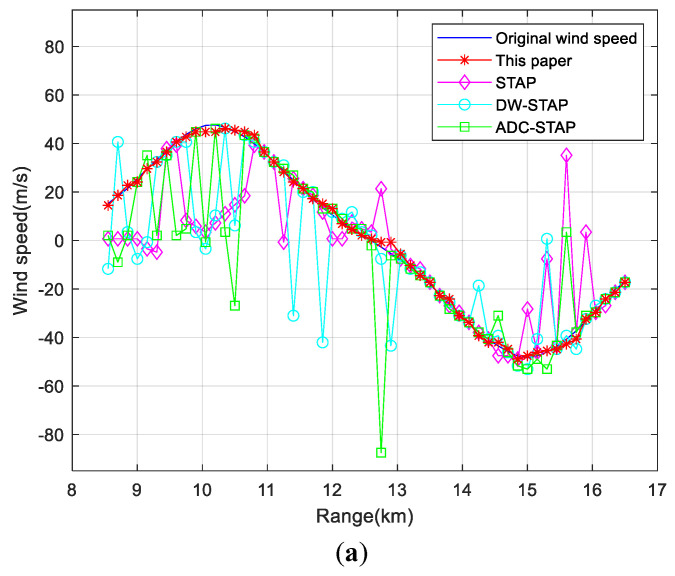

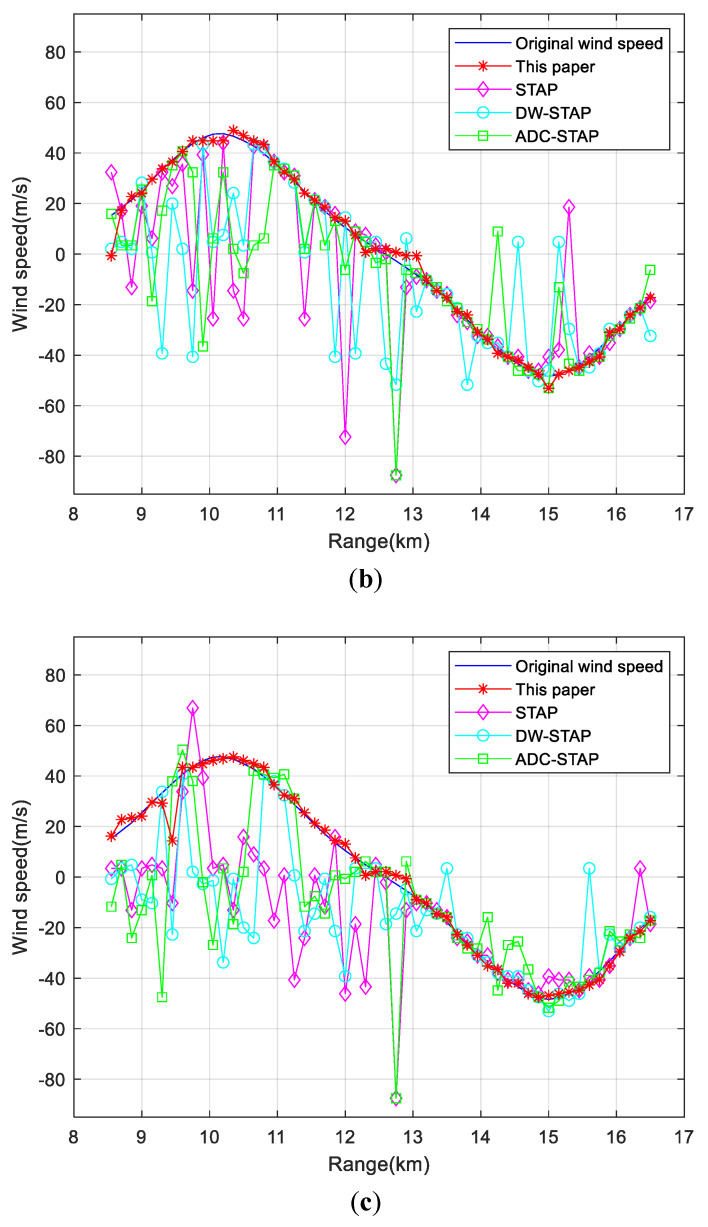
Comparison of the wind speed estimation results under different CNR conditions. (**a**) 40 dB (**b**) 50 dB (**c**) 60 dB

Figure 7 shows the comparison of the wind speed estimation results of low-altitude windshear by each method under different clutter-to-noise ratio conditions. Figure 7a–c show the wind speed estimation results when the ratio is 40, 50 and 60 dB, from which it can be seen that the wind speed can still be estimated accurately when the ratio increases.

Table 2 shows the comparison of the root-mean-square error of wind speed estimation for each method under different spurious noise ratio conditions. From the table, it can be seen that the increase in the clutter-to-noise ratio has a large effect on the RMS error of STAP, DW-STAP and ADC-STAP methods, while the RMS error of the methods in this paper are less affected by the clutter-to-noise ratio.

**Figure 8:**
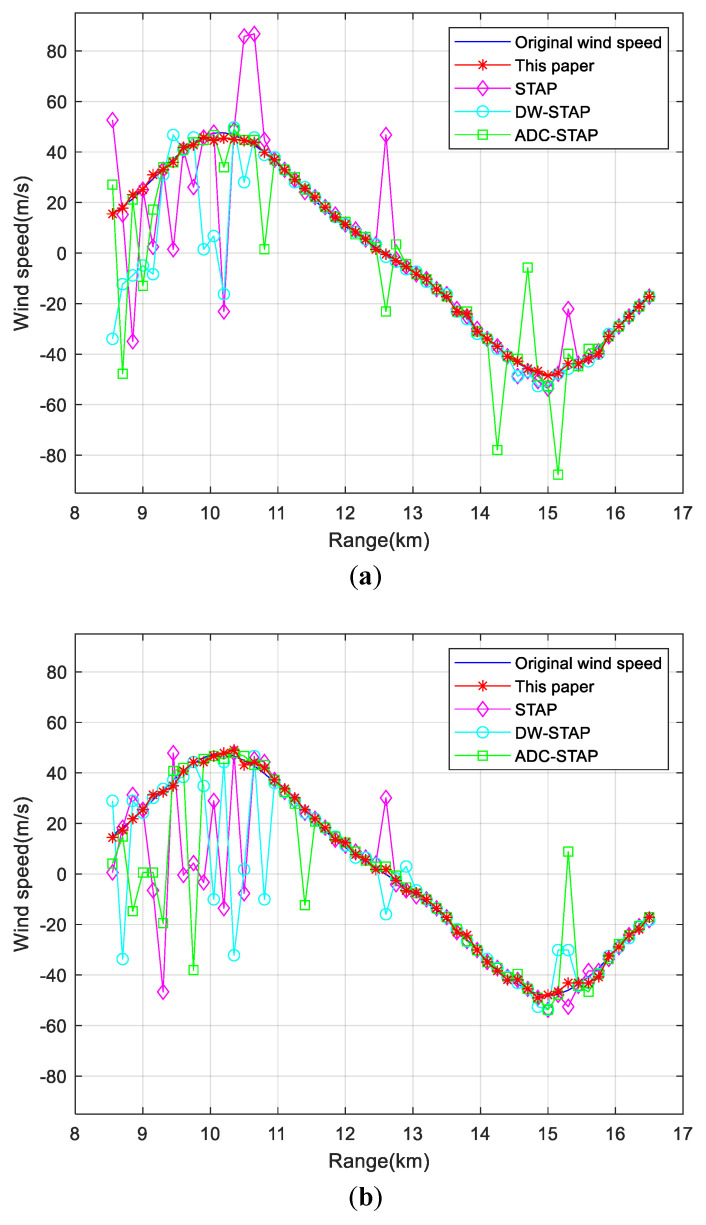

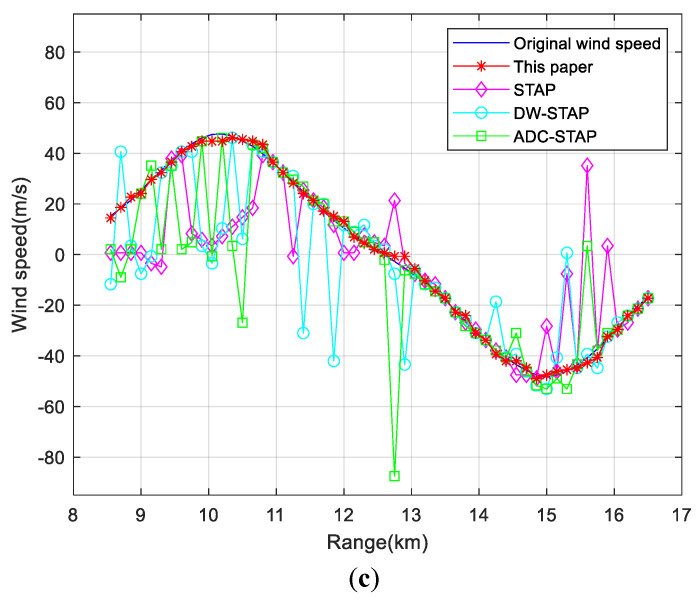
Comparison of the wind speed estimation results under different PRF conditions. (**a**) 5000 Hz (**b**) 6000 Hz (**c**) 7000 Hz

Figure 8 shows the comparison of wind speed estimation results of each method for low-altitude windshear under different PRF conditions. Figure 8a–c show the wind speed estimation results at PRF of 5000, 6000 and 7000 Hz, from which it can be seen that the method in this paper can still estimate the wind speed accurately at different PRF.

Table 3 shows the comparison of the root-mean-square error of wind speed estimation for each method under different PRF conditions. From the table, it can be seen that the change of PRF has a large effect on the root-mean-square error of STAP, DW-STAP and ADC-STAP methods, while the root-mean-square error of the methods in this paper are less affected by PRF.

## 5. Conclusions

For the problem that the useful signal is affected by strong ground clutter when detecting low-altitude windshear, a method for estimating low-altitude windshear wind speed in the fuselage frustum conformal array system is proposed. The method introduces the fuselage frustum conformal array and analyzes the essential reasons for the difficulty of clutter suppression in the fuselage frustum conformal array. Then, it uses a four-dimensional frequency domain compensation method to combine the phase compensation of the echo data of each distance unit in the Doppler domain and the three-dimensional space-frequency domain. After that, it estimates the clutter covariance matrix from the compensated echo data samples and constructs a space-time adaptive processor applicable to the low-altitude windshear target. Finally, it extracts the maximum Doppler value of each distance unit after adaptively suppressing the ground clutter to achieve the accurate estimation of wind field velocity. The simulation results show that the method can effectively suppress the ground clutter and accurately estimate the wind speed in the fuselage frustum conformal array system.

## Figures and Tables

**Table 1:** Aircraft and radar simulation parameters.

Parameters	Parameter Value	Parameters	Parameter Value
Height of aircraft (m)	600	Number of rows	4
Speed of aircraft (m/s)	87.5	Number of columns	12
Radius of the bottom surface of the frustum (m)	0.6	Sampling pulse number	32
Height of Frustum (m)	0.3	Pulse repetition frequency (Hz)	7000
Inclination of frustum (°)	45	Signal to Noise Ratio (dB)	5
Center of circle angle (°)	22.5	Clutter to Noise Ratio (dB)	40

**Table 2:** Comparison of RMS error of wind speed estimation results under different CNR conditions.

CNR	Wind Speed Estimation Method	RMS Error (m/s)
40 dB	STAP	21.0683
	DW-STAP	19.8742
	ADC-STAP	21.1224
	This paper	1.5196
50 dB	STAP	27.4548
	DW-STAP	26.5530
	ADC-STAP	24.0274
	This paper	2.9997
60 dB	STAP	28.4267
	DW-STAP	28.0813
	ADC-STAP	27.11929
	This paper	3.6221

**Table 3:** Comparison of root-mean-square error of wind speed estimation results under different PRF conditions.

PRF	Wind Speed Estimation Method	RMS Error (m/s)
5000 Hz	STAP	18.4618
	DW-STAP	16.5961
	ADC-STAP	15.6833
	This paper	1.0402
6000 Hz	STAP	20.0491
	DW-STAP	18.2206
	ADC-STAP	17.7085
	This paper	1.3193
7000 Hz	STAP	21.0683
	DW-STAP	19.8742
	ADC-STAP	21.1224
	This paper	1.5196

## Data Availability

Not applicable.
